# Life-course perspectives of milk consumption among young Norwegian women and their knowledge of milk as a source of iodine: a qualitative study

**DOI:** 10.29219/fnr.v65.7758

**Published:** 2021-12-14

**Authors:** Sigrun Henjum, Synne Groufh-Jacobsen, Inger Aakre, Laura Terragni

**Affiliations:** 1 Department of Nursing and Health Promotion, Faculty of Health Sciences, Oslo Metropolitan University, Oslo, Norway; 2 Department of Nutrition and Public Health, Faculty of Health and Sports Science, University of Agder, Kristiansand, Norway; 3 Department of Seafood and Nutrition, Institute of Marine Research, Bergen, Norway

**Keywords:** food choice trajectories, food practices, milk consumption, iodine, life-course perspective, Norway, young women

## Abstract

Mild to moderate iodine deficiency has been found among young Norwegian women of reproductive age. In Norway, cow’s milk is the main source of iodine; however, milk consumption is decreasing, particularly among young women. This study aimed to investigate milk consumption practices in young Norwegian women and their attitudes toward milk consumption from childhood to young adulthood in a life-course perspective and their knowledge of milk as a source of iodine. Convenience sampling was used to recruit 30 bachelor students (women, 18–25 years old) from five different study programs. Interpretative phenomenological analysis (IPA) was used to interpret milk consumption practices from a life-course perspective. Five focus group interviews were conducted using a semistructured interview guide. The transcribed interviews were coded according to emerging themes related to milk consumption practices and turning points. Milk consumption practices were dynamic and changed over time and were influenced by several factors: family traditions, school milk subscription, friends and social media, social acceptance, availability, price, and attitudes toward health and the environment. Young women tend to be in a phase of life in which milk is not part of their food practices. Most of the women were not aware of the consequences of omitting milk from their diet and had limited knowledge of iodine and how to secure adequate dietary iodine intake. Awareness of possible consequences of omitting milk from the diet should be promoted along with information on how to secure adequate iodine intake.

## Popular scientific summary

- Iodine is important for fetal growth and iodine deficiency has been found among young Norwegian women of reproductive age.
- Milk is an important source of iodine. Young women reduce their milk consumption or omit milk from their diet for reasons related to attitudes toward health and environment, convenience and price. Initiative aimed at increasing awareness about iodine sources are needed.

In Norway, there is a growing concern regarding decreased milk consumption among young women since milk is the major source of iodine in the Norwegian population (1–3). Iodine is a critical nutrient for women of childbearing age to secure optimal fetal growth and development (4). Milk, dairy products, and fish are the main iodine sources in countries without mandatory salt iodization, including Norway. In Norway, approximately two-thirds of the dietary iodine intake comes from cow’s milk and dairy products (due to iodine fortification of the cow fodder), while the consumption of lean fish with a high concentration of iodine has decreased (5). Thus, individuals who exclude milk and dairy products will be at high risk of insufficient iodine intake. The majority of the plant-based milk alternatives that are on the rise in the Norwegian market are not iodine enriched (6). In Norway, mild to moderate iodine deficiency has been found among pregnant and lactating women (1, 7) together with limited knowledge of iodine and health (8–10). Yet, little attention has been given to investigating young women’s attitudes toward milk, their milk consumption practices, and their knowledge about the potential health consequences of omitting milk from the diet (1–3, 7).

## Milk in the Norwegian Society

In Norway, milk has traditionally been and still is a central component of most people’s diet, and it holds a strong position in Norwegian nutrition policies (11). The important role of milk in the Norwegian diet can be traced back to the 1920s, after World War I. Milk was seen as a nutritional ‘miracle food’ and a national symbol in a country where – at that time – poverty, undernutrition, and child hunger were common. Since the interwar period, milk has been promoted in Norwegian nutrition policies due to its beneficial impact on health (12, 13). By the 1930s, the official Norwegian recommendation was to consume one liter of whole-fat milk per person per day (14, 15). The relevance of milk in the Norwegian diet remained strong also after critiques in the 1960s, when the mortality rate of cardiovascular disease increased dramatically, and concerns gradually raised regarding dietary fat. Current recommendations from the Norwegian Directorate of Health are three portions of skimmed or low-fat milk or lean dairy products per day for the general population (16). Subsidized milk has been offered since the 70s for children participating in the ‘school milk subscription’; today, milk is still an important component during lunch in Norwegian primary and secondary schools.

## Practices of milk consumption in a life course perspective

Eating and drinking practices are shaped alongside everyday activities that take place in families, at work, and in schools (17). Norms, expectations related to what is appropriate to eat, tend to be contextual and vary accordingly to different circumstances. Food and drinking practices tend to change over time, as people make changes in their lives and daily routines and develop new preferences, values, and concerns about the food they eat (18, 19).

The life-course perspective is a framework that provides an understanding of how food and drinking practices change along the life course (20). This perspective takes into consideration contextual aspects related to societies as well as aspects related to an everyday individual’s life. ‘Food Trajectories’ and ‘Turning Points’ are central key concepts in the life-course perspective (20). The trajectory for consumption of specific food can go through transitions or ‘turning points’ due to events in an individual life or society. The life-course perspective has been adopted by studies investigating food consumption, providing interesting insights in understanding how current food or eating practices involve past experiences as well as current values and expectations about future outcomes and possibilities (21–23).

By adopting a life-course perspective, this study aims to explore how milk consumption practices change across the life-course of young Norwegian women, how phases of transition shape their use of milk, and their knowledge of milk as a source of iodine. Knowledge about milk consumption practices among young women can contribute to understanding the possible reasons for the decrease in milk consumption of this group and to develop nutrition communication initiatives to increase awareness about the consequences of omitting milk from the diet.

## Materials and methods

### Study design

This study had a qualitative interpretative research design (IPA), which aims to give insights into how a person, in a given context, makes sense of a given phenomenon (24). This approach appears to be particularly suitable when informants are asked to reflect upon their personal experiences, such as food practices, like in this study. Although IPA is mostly employed in interviews, the use of IPA in focus groups interviews is increasing (25). Group discussion may, in some situations, elicit more reflection than a one-to-one interview (25). As the experiences of milk consumption practice through the life-course may be a topic not many have reflected much upon, bringing it up in focus groups may facilitate the recollection of memories and the process of making sense of transition in milk consumption practices during life.

### Recruitment

By convenience sampling (26), participants were recruited from two universities located in Oslo, during August and September 2018. Students were recruited from five different study programs, including economy (E), psychology with an emphasis on behavior analysis (P), project-design (PD), social work and nursing (SN), and public health nutrition (PHN). Students were contacted at the study site and invited to participate in focus group interviews. Eligibility criteria included to be a female student, have an age between 18 and 25 years old, and not having children. Five focus group interviews with young female students from each study program were performed. Each focus group consisted of 5–7 participants. This number is considered appropriate to elicit common discussion while keeping the groups small enough to create a comfortable environment (27). Each participant was rewarded with a gift card. The focus group interviews were moderated by a trained researcher and lasted from 60 to 90 min. A semistructured interview guide (Supplemental 1) based on the life-course perspective of food practices was developed (21). Probes were used to promote further reflections and exemplifications from participants.

### Analysis

Each focus group interview was digitally recorded and transcribed by the second author. The first author compared the transcripts with the audiotapes for quality control and then imported them into NVivo (software version 10; QSR International, Burlington, MA). Focus groups transcriptions were read several times by the first and last authors to be familiar with the data (24). Using an inductive approach, the two investigators independently analyzed the transcripts to identify emerging themes and subthemes related to the milk consumption practices in a life-course perspective (28). An inductive approach implies that themes identified are strongly linked to the data themselves and not to a previous theoretical framework. For each focus group, we created a ‘milk consumption practice trajectory’ and compared it with the findings of other focus groups to identify similar patterns and differences. Transition phases were identified by looking at when participants talked about changes in milk consumption practices (for instance, drinking less milk after moving out from home). The ways practices and the meaning of milk changed during the life course emerged by considering how participants talked about milk. Initially, a large number of codes emerged from the data. These were subsequently grouped into overreaching subthemes (for instance, all codes containing forms of obligation or pressure of drinking milk, where grouped in the subtheme ‘Milk as a must’). The analysis ended when no new themes were identified, achieving what has been defined as data saturation (29). Recurring themes and subthemes emerging from the analysis are shown in Table 1.

**Table 1:** Milk consumption practices in a life-course perspective: Themes and subthemes emerging from the focus group interviews

a) Milk consumption practices during childhood	Milk as ‘family’
	Milk as ‘tradition and health’
	Milk as ‘a must’
	Milk as something everyone drinks
b) Milk consumption practices during adolescence	Milk as childish
	Milk as not healthy
	Milk and skin health
	Milk and influence by friends and classmates, social acceptance
c) Milk consumption practices during young adulthood	Milk in new life stages like moving away from home
	Milk as expensive and inconvenient to use
	Milk practices influenced by media
d) Milk consumption practices in the future e) Awareness about the consequences of omitting milk from the diet	Milk important for future children
	Milk and role models for own children
	Lack of knowledge of iodine sources
	Milk not important for health

### Ethics

The young women were informed about the study purpose, and those willing to participate gave an informed written consent before the start of the study. This study was conducted according to the guidelines in the Declaration of Helsinki and was approved by the Regional Committee for Medical and Health Research Ethics in Norway (2015/1845).

## Results

Five focus group interviews with young women, all bachelor students (18–25 years of age), were held. The young women had Norwegian origin, except one of Indian origin. Of the 30 participants included in the study, 10 participants reported regular consumption of milk, while the majority reported to rarely consume milk or having omitted milk from their diet. Quotes from the focus group discussion are presented using abbreviations (Study program and respondent number).

### Milk consumption practices during childhood

Most of the young women expressed that milk was an important part of their diet during childhood. The young women reflected on how milk was a traditional part of an everyday routine during childhood as an everyday drink, at breakfast, lunch, dinner, and when they were thirsty. Some of the young women mentioned that they were told to drink milk by their parents to grow strong and healthy, and that milk was almost always available on the table during meals.

P3: *I drank a lot of milk during my childhood; everyone drank milk*.

P, all: *Yes*

P2: *I also grew up with the fact that everyone liked milk and should drink milk. You get milk or water, or nothing else. I remember that when I was younger, I used to drink milk. My dad drank milk and my sister as well, while my mum, she drank chocolate milk because she did not like plain milk. But she drank milk to be a good role model for her children*.

P4: *We always had a lot of milk at home because everyone drank milk (…), Mom normally drank milk, and she loves milk too. Dad is also very fond of milk*.

P1: *My Father brings two cartons of milk to work*.

P3: *Every day?*

P1: *For lunch*.

Milk was available through a school milk subscription in Norwegian schools. Most participants drank milk at school through the daily milk subscription. The interviews indicated that in primary schools, milk subscription was considered very popular, and the young women reflected on the importance of fitting in.

Interviewer: *Did any of you have milk subscription in primary school?*

P, all: *yes.*

P3: *I felt that it was a group pressure, like a drinking pressure (laughs). You were not cool if you did not have the milk subscription somehow*.

P4: *… or those who had strawberry milk, they were not cool at all*.

P1: *I had a period where I wanted to be different from everyone else. I refused to do the same as everyone else did… So I did not want to drink milk just because everyone else did*.

However, not all young women experienced milk in childhood as caring and family bonding. For some, milk brought back negative childhood memories of lukewarm milk with lumps and bad smell and memories of being forced to drink up the glass of milk served at meals.

PHN1: *As a child I was not very fond of drinking milk. My brother, yes (…) he poured it down, but I (…) while my dad kept filling up my glass, I kept saying, thank you. Thank you. But he just kept filling up. I couldn’t leave the table until I had drunk this one glass of milk. In my family, milk was important*.

### Milk consumption practices during adolescence

A first shift in milk consumption practices seemed to occur during adolescence when attendance to junior high began (14 years). Some stopped drinking milk because they felt that it was associated with being childish. From the beginning of junior high, the young women reported that they no longer had school milk subscription, either because their junior high schools did not offer it or their parents allowed them to decide whenever they wanted to subscribe to school milk or not. The young women said that they were tired of drinking milk and dropped the subscription, choosing to buy other drinks in the school canteen instead, like juice or soda. The young women mentioned that ‘the opportunity to decide by themselves’ might have been a factor for omitting milk from their diet.

SN4: *I think maybe one can get a little tired (of drinking milk) too. Because I think when you drink some milk every day for ten years, and it wasn’t just in school*.

During the focus groups, participants reflected upon the fact that adolescents are also easily influenced by friends and classmates as they are striving to be accepted and to fit in. Subtheme emerging within this theme was milk as ‘Social acceptance’, as the participants explained how it was important to do the same as their friends and that their friends had stopped drinking milk.

PD3: *Before, everyone drank milk, but now there is very little of it in general. All my friends have also stopped drinking milk*.

Young consumers today deal with an enormous amount of health and nutrition information often claiming that milk is not healthy as it contains hormones and antibiotics or that it is not important for health. The young women participating in the focus groups were, in general, concerned about eating healthy and experienced that their milk consumption was affected by the information they got from media, which questioned the idea of milk as healthy and not good for their body.

PHN2: *In junior high school, milk was always available in the canteen if you wanted buy it, but I do not recall anyone who actually said (…) looking forward to lunch, I think I will buy a milk*.

PD4: *There has been a lot of media attention claiming that milk is not healthy*.

PHN2: *I don’t think milk is necessary. I have seen documentaries and read a lot about it and I have heard that there are hormones in the milk that make you addicted. That is why the calf keeps going back to his mother to drink more. It’s not something I want to make myself addicted to (…) I notice that… I noticed that when I drank milk, I kept wanting more*.

Concerns about animal welfare also emerged. Documentaries like ‘What the health’, ‘Cowspiracy’, and ‘Forks over knives’, were reported to influence their thoughts about milk, and some had concluded that drinking milk was not ethical both because of the living conditions of the animals and because of environmental reasons.

SN3: *‘I have heard that in Norway, we are at the top of milk drinking in the world, but also on the top for bone fractures, osteoporosis and things like that. … I’m probably one of those who thinks that milk in a way belongs to the calf. I do not know. But I feel that you either really love or hates milk. I have probably been influenced by documentaries. They talk much about meat industry and milk production*.

Skin health was a concern for many of the young women, and some referred to have heard that they could get pimples or acne from milk. Others said that they felt bloated and got a stomachache from drinking milk. They felt better when they reduced or stopped drinking milk, and they felt more energetic and had less pain.

PD5: *In my teens, I was told that milk was not good for my skin*.

PD1: *Yes*

PD5: *That you got pimples and such things, so I was like ‘nop, I do not want to drink milk. Then I stopped’*.

PHN2: *I drank milk during childhood. I stopped in secondary school because of acne; I feel I am affected when I eat many milk products. That I get dirty skin from it*.

### Milk consumption practices during young adulthood

Another significant ‘turning point’ in milk consumption came when the young women moved away from home. Milk was no longer available all the time as in their childhood homes. They had to buy the milk themselves. Some women explained that they did not want or could not spend their money on milk because it was more expensive than soda and juice, and they were not able to drink the milk before it expired. The young women reflected upon the impact of availability, as many did not prioritize buying milk on student budget.

PHN4: *I started to drink less milk when I moved away from home, because then I had to buy the milk myself. (….). I didn’t bother spending money on it. It’s cheaper with juice and stuff like that*.

SN2: *In addition, it expires rapidly (laughs), so then it is sort of … it becomes like that I rarely buy milk then. Because I know, I cannot drink it up*.

Almost none of the young women consumed a glass of milk when they were thirsty or together with a meal like they did during childhood. The young women reported that they had replaced milk with water or soda when they were thirsty. However, the young women reported that they still drank milk on special occasions, like when they visited their parents or grandparents.

SN2: *I drink milk when I visit my parents, but since I live alone it is seldom that I drink milk*.

Despite the limited use of milk as a beverage, many of the young women reported that they added some milk to their coffee and used it in food preparation, like for porridge, in sauces, or with cereals.

SN2: *I buy milk for food preparation, such as when I make a sauce or porridge*.

Many plant-based alternatives for milk have emerged in the last few years, like oat, soya, and rice milk, with information that they are healthier than cow’s milk. The products are considered more ‘trendy’ or a better alternative for those who feel discomfort by drinking milk. Furthermore, those who experience negative effects when consuming milk had replaced cow’s milk with plant-based milk alternatives.

SN6: *It has become very popular having a diet with focus on body and eating healthy and all that… People who follow these diets also tells that it is not okay to drink milk for some reason. I don’t know why they tell such thing, but it has become very popular with soy milk, oat milk and almond milk*.

Not everybody held negative associations with cow’s milk. Some women had many positive experiences with milk and drank milk daily. Their perceptions of milk were related to a healthy and strong body and exercise. Some of the young women mentioned that they preferred cow’s milk, not milk alternatives like oat milk or almond milk, because of the taste and the price.

SN2: *I love to drink milk. I drink milk daily for breakfast… I love milk. I kind of… I can drink it before I go to bed. It is a must to drink milk during the day. I think ice-cold milk is delicious*.

SN1: *You get big and strong if you drink milk. I feel pretty healthy, and I drink a lot of milk*.

PHN6: *I have tried both almond and oat milk, but I found plain milk tastes much better, and it is much cheaper too*.

### Milk consumption practices in the future

When the young women were asked if they would give cow’s milk to their children, the responses went in both directions. Some mentioned that milk consumption is important during pregnancy and for children during their first years of life. Interestingly, some reported that they would start to drink milk again if they had children of their own, to be a good role model.

Interviewer (1): If you had a small child now, would you have given your child cow’s milk?

SN1: *Yes*.

SN5: *Absolutely*.

SN3: *No*.

SN1: *I would have had milk in the fridge. It’s probably just something I would have done because my mom did it*.

SN2: *I think that I would start buying milk regularly*.

SN1: *I think it’s important the first few years, maybe … if it’s actually true what they say about the skeleton and all that*.

SN2: *Yes*.

SN5: *I would probably have started drinking some milk just to be a good role model. Just force myself to have a glass a day somehow*.

Interviewer (2): *Would you give cow’s milk to your future children?*

E3: *Yes*.

E2: *Yes, I would have forced my child… you must have milk (…). I think I would have made it as a habit for my child somehow. Maybe that they had to drink milk for breakfast somehow, and when they got older it will be their own choice*.

E4: *I don’t think I would do it, because I don’t like milk*.

E2: *But would you not even try to… to get your child to…?*

### Awareness about the consequences of omitting milk from the diet

In our interviews, the young women were asked if they knew what iodine was. Most of the women admitted that their knowledge of iodine was very limited and some even mentioned that they had never heard about iodine before.

Interviewer: Have any of you heard about iodine before?

E1: *Yes*.

E3: *No*.

E2: *What?*

E3: *Iodine, what is that?*

E1: *I don’t know. I cannot explain what it is*.

E4: *Is there something you need like a vitamin or something?*

E1: *Yes*.

E3: *I have no idea*.

The young women were also asked if they knew about any dietary iodine sources. The young women identified table salt as a source of iodine, as they had seen the word on the table salt. Only a few participants had heard that milk and fish contained iodine, which are the main iodine sources in the Norwegian diet.

P3: *I don’t know, I have no idea (laugh). Do you know what iodine is? [speaking to the others] I don’t even know about any iodine food sources. I’m totally lost here*.

The young women were generally not aware of iodine’s functions in the body.

PHN2: *I think there are only a few people who really know what iodine does in the body. To be perfectly honest, I have never particularly reflected upon my iodine intake*.

PD4: *Yes, I have heard about it, as an ingredient, but I do not know what it is. Neither do I know what it is good for or anything else*.

Given this lack of knowledge of what iodine is and dietary iodine sources, it is not surprising that most of the young women had not thought much about the health consequences of omitting milk from their diet. Many knew that milk is an important source of calcium and some mentioned bone fracture and osteoporosis as consequences of excluding milk from the diet. An interesting finding from our interviews was that young women did not seem to be particularly worried about omitting milk from their diet.

SN3: *It is not like you die or do not survive without milk, or that something bad happens if one does not get milk products. It is not a necessity*.

PHN2: *I do not think that it is a problem if you do not drink milk. There are so many countries where people do not drink as much milk as in Norway. They manage anyway…*

During the interviews, the importance of iodine was discussed, some of the young women started to be worried about their lack of information about having enough iodine in their diet and wished that this information was provided to them.

PD3: *I really feel a little cheated because I have never really heard anything about iodine …. You are told about the importance of other things like vitamin D, calcium and all these nice things. But iodine is actually kind of forgotten*.

## Discussion

Wanting to address the worrying low iodine status among young women in Norway, this study investigated milk consumption practices in a life-course perspective focusing on how events and experiences have shaped food practices in a sample of young Norwegian women in the Oslo area. Our study indicates that milk consumption is dynamic and changes over time. Most of the young women experienced transitions and turning points that influenced their milk consumption. We found that the consumption of milk during childhood was greatly influenced by the positive societal attitudes toward milk, family traditions, and school milk subscriptions. The first transition in milk consumption occurred during adolescence. The young girls seemed to distance themselves from milk consumption as it was associated with childish food habits, and new beverages became more popular among peers. The main turning point occurred, however, during young adulthood when the young women left home. In this new phase of their life, factors such as availability, price, convenience, and attitudes toward their health and the environment became increasingly important. Coupled with a lack of knowledge about iodine and its relevance for young women, this may contribute to explain why milk consumption is low within this group. Milk consumption practices in a life-course perspective will be discussed below.

### Childhood

Food choice trajectories are developed within a specific situational and historical context (20). Most of the young women grew up in a society, as Norway, where milk had a strong position both as a nutritional source and as a national symbol of health and strength (11). It is, therefore, not surprising that the young women in our study experienced that milk consumption at home and at school was an important part of their diet during childhood, and that children were expected to consume and like the taste of milk. Milk was embedded in their daily routines and was an important component of the practice of ‘being a family’ or ‘being a proper child’ (30).

### Adolescence

As the young women entered junior high, a transition in their milk consumption occurred. Adolescence is a period of time during life-course, in which individuals want to make their own decisions, often opposite of what they have been thought by their parents. During adolescence, food consumption becomes a way to distance oneself from childhood and parents (31). Food and drinking practices are used to build new identities and to signal belongings with friendship and peer norms (32, 33). In our study, young women reported that they experienced getting tired of milk, and that they were more concerned about social acceptance from their classmates and friends and their perceptions of milk. Similar findings have been found among adolescents in Norway, showing that milk drinking was abandoned in junior high and substituted with other beverages (34, 35).

### Young adulthood

The most significant turning point in milk consumption occurred, however, when the young women left home. It is important, in this regard, to point out that in Norway, young people leave their parental household by the median age of 19 years, compared to the overall estimated average age of young people in Europe at 26 years (36). According to a smaller study, only one in five Norwegian students younger than 22 years of age still live with their parents (37). In our study, a large part of the respondents had left home and lived either alone, in shared apartments, or with a partner. As previous studies have pointed out, this transition creates new food consumption practices. For young students, this implies that they must provide their own meals, often with a small budget, a tight time schedule, and limited cooking skills (38). Price and convenience were aspects that also emerged among the young women, as they were likely to skip buying milk because it was expensive to buy, and it expired before they were able to use it. The shift in milk consumption reported in our study may not only due to practical or economic reasons but also reflects changes in attitudes toward milk in society (38). While their parents had grown up in a society with strong traditions and positive values related to milk consumption, the young women were more exposed to contested ideas about milk, both in terms of health and sustainability derived from a globalized culture.

Differently from the generation of their parents, which was provided nutrition information by few and highly trusted sources – as the Norwegian Nutrition Council – the new generations are influenced by a plurality of communication sources (e.g. advertisements, influencers, celebrities, and documentaries) (39). With almost unlimited access to unregulated information sources, some people seem to feel that the verification of food sources and its quality is their own personal responsibility and turn to the media for help (40). Yet, the quality of health- and nutrition-related messages in the media is perceived as contradictory and confusing and often not necessarily the most credible (41).

### Milk consumption practices in the future

Our study indicates that there are several reasons that can contribute to explaining why milk is a less consumed food item among young women. Interestingly, also among those who have significantly reduced or abandoned milk consumption, there are some who consider introducing milk to their children. This indicates again that food choice trajectories must be understood as dynamic and related to negotiations among different values, opportunities, and roles in different phases of life. As milk represented positive emotions of care, comfort, and healthiness during their childhood, some women, imagining themselves in their role as a mother and as responsible for providing their children and their family with a healthy diet, stated that they would reintroduce milk. This attitude toward milk provides an interesting insight into the definition and redefinition of food during lifetime in the categories of ‘edible’ and ‘not edible food’, or ‘pure’ and ‘polluted’ (42, 43). Milk, as this study illustrates, from being a fundamental part of diet and a symbol of ‘pureness and healthiness’ as it was during childhood becomes a more contested food for practical, cultural, health, and ethical reasons during young adulthood, but is likely to regain importance in a later phase of life.

### Awareness about the consequences of omitting milk

Young women’s low iodine intake can have consequences for their future children, who can be susceptible to the adverse effects of iodine deficiency (44). According to a recent study on iodine knowledge (3), approximately 40% of young Norwegian women had little knowledge of dietary sources of iodine and about the health implications of omitting iodine-rich sources from their diets. Our study supports these findings and raises concerns as it reveals the paradox of young women growing up with milk and planning to provide milk to their children but reduce their milk consumption in the very phase of life where the intake of nutrients assuring adequate iodine intake is of utmost importance.

This study has some strengths and limitations. The use of a life-course perspective to explore milk consumption practices allowed us to consider the intersection between personal and societal events, such as the role of milk in Norwegian society and during the childhoods of young women as well as the shifts in societal attitudes toward milk. Focus groups facilitate sharing of experiences, memories, and awareness. A possible limitation of the study is the fact that all the young women were university students, and that this study was conducted in an urban context, in Oslo. One may expect that in rural areas, traditional values related to milk consumption may prevail. However, Oslo tends to attract students from all over the country, including students from rural areas. Moreover, the variation of the study programs from which the students were selected may have contributed to the recruitment of young women with different backgrounds and knowledge of nutrition. Participants from PHN may be more informed about nutrition-related matters as compared to students from other disciplines. However, given the fact that they were first year’s students, we assumed that their knowledge was limited.

## Conclusions

We found that milk consumption practices changed during life-course, which influenced by several factors, such as family traditions, school milk subscription, friends and social media, availability, price, and attitudes toward health and environment. Most of the young women were in a phase of their life when attitudes toward milk were negative or ambivalent, and milk consumption was not part of their daily routines. Our findings provide an understanding of possible reasons for the reduction of milk consumption among young Norwegian women. Our study further indicate the need of promoting awareness of the consequences of omitting an important source of iodine as milk from young women’s diet and develop strategies for assuring adequate iodine intake.

## New contribution to the literature

1. Provides insight into young women’s attitudes toward milk and milk drinking practices, as milk is the main source of iodine in Norway. Iodine is of relevance for fetal growth and child cognitive development. Previous studies have indicated that pregnant and lactating women have low iodine status. It is important to understand why young women tend to reduce their milk consumption in a phase of their lives when assuring an adequate intake of iodine is of utmost importance.
2. Highlights the relevance of adopting a life-course perspective for understanding factors that have an impact on their dietary choice and the importance of social acceptance in young adulthood.
